# Enhancing Endosomal Escape of Transduced Proteins by Photochemical Internalisation

**DOI:** 10.1371/journal.pone.0052473

**Published:** 2012-12-21

**Authors:** Kevin Mellert, Markus Lamla, Klaus Scheffzek, Rainer Wittig, Dieter Kaufmann

**Affiliations:** 1 Institute of Human Genetics, University of Ulm, Ulm, Germany; 2 Institute for Organic Chemistry III/Macromolecular Chemistry, University of Ulm, Ulm, Germany; 3 Division of Biological Chemistry, Biocenter Innsbruck Medical University, Innsbruck, Austria; 4 Institut für Lasertechnologien in der Medizin und Messtechnik (ILM) an der Universität Ulm, Universität Ulm, Ulm, Germany; Oak Ridge National Laboratory, United States of America

## Abstract

Induced internalisation of functional proteins into cultured cells has become an important aspect in a rising number of *in vitro* and *in vivo* assays. The endo-lysosomal entrapment of the transduced proteins remains the major problem in all transduction protocols. In this study we compared the efficiency, cytotoxicity and protein targeting of different commercially available transduction reagents by transducing a well-studied fluorescently labelled protein (Atto488-bovine serum albumin) into cultured human sarcoma cells. The amount of internalised protein and toxicity differed between the different reagents, but the percentage of transduced cells was consistently high. Furthermore, in all protocols the signals of the transduced Atto488-BSA were predominantly punctual consistent with an endosomal localisation. To overcome the endosomal entrapment, the transduction protocols were combined with a photochemical internalisation (PCI) treatment. Using this combination revealed that an endosomal disruption is highly effective in cell penetrating peptide (CPP) mediated transduction, whereas lipid-mediated transductions lead to a lower signal spreading throughout the cytosol. No change in the signal distribution could be achieved in treatments using non-lipid polymers as a transduction reagent. Therefore, the combination of protein transduction protocols based on CPPs with the endosomolytic treatment PCI can facilitate protein transduction experiments *in vitro.*

## Introduction

In cell culture experiments, the transduction of functional proteins into cells has become an alternative strategy with respect to transient (or stable) transfection of the cells with specific gene expression vectors. Current protocols use different strategies to overcome the cellular membrane, essentially basing on cell penetrating peptides, lipid shuttle systems, non-lipid (cationic) polymers or endosomolytic reagents.

Starting with the discovery of the protein transduction domain of the TAT protein from HIV, various short peptide sequences have been found to be able to enter living cells [1]. These so called cell penetrating peptides (CPPs) have also been shown to be capable of transporting attached cargos into a wide range of cultured cell types [2]. Up to now, it is still under debate whether the internalisation pathway of CPPs and their cargo is energy dependent [1], [3] or not [4]. At least for CPPs transporting high molecular weight cargo, like proteins, it seems to be widely accepted that different forms of endocytosis are the main uptake pathway [2]. Lipid shuttle systems and non-lipid polymers are used for transduction of proteins in cultured cells as well [5], [6]. Overcoming the endo-lysosomal entrapment remains the major problem in published transduction protocols of proteins.

Several strategies were tested to solve this problem. Co-treatments with chloroquine for example showed that the amounts of applied chloroquine needed to disrupt endosomal vesicles were already in the cytotoxic range [7], [8]. Another method to induce endosomal disruption is the usage of the NH_2_-terminal 20 amino acid peptide derived from the influenza virus hemagglutinin-2 protein (HA2) either as a co-incubation reagent or covalently bound to the transduced protein, which may be combined with the transduction capacity of CPPs [9], [10]. An additional technique to disrupt endosomes is the photochemical internalisation (PCI) [11], [12]. During this treatment, cells are preincubated with a photosensitizer, which initially integrates into the cellular plasma membrane and further distributes to endo-lysosomal membranes via endocytosis. An exposure of the treated cells with light of a photosensitizer specific wavelength results in disrupting especially the endo-lysosomal membranes by generation of reactive oxygen species (ROS) in the membranes [12]. This PCI treatment has been tested successfully in endosomal release of gelonin in tumor cells *in vitro* and *in vivo* [13], [14]. A combination of PCI with protein transduction has been described very recently, but only for transductions via TAT-fusions or a lipid shuttle system [15], [16]. In this study, we provide a comparative analysis of the efficiency of a combined application of PCI and common methods of protein transduction.

We wanted to find out whether endosomal entrapment can be overcome with non-covalently bound transduction systems using a combination of transduction and photochemical internalisation. Furthermore we wanted to explore the influence of differing internalisation strategies on both protein transduction and endosomal release of the internalised protein. To investigate this issue, we transduced a fluorescent protein (Atto488-BSA, 67 kDa) as cargo into human sarcoma cells using different commercially available transduction reagents based on CPPs, lipid shuttle systems, non-lipid (cationic) polymers and an endosomolytic reagent. In the following study, we performed PCI treatments with the protein transduced cells and compared signal spreading throughout the cytosol.

## Methods

### Cell Culture

The NFS4 cell line was established by us from an excised human neurofibrosarcoma of a 24 years old Neurofibromatosis Type 1 patient of European descent, the human fibroblasts (FP1) were obtained from the prepuce of a 3 year old healthy donor of European descent [17]. All main experiments were carried out in the neurofibrosarcoma cells. Fibroblasts were only used to confirm that protein uptake in vesicles is not a special characteristic of the neurofibrosarcoma cells. The research carried out was in compliance with the Helsinki Declaration. The participants provided their written informed consent to participate in this study and the experiments were approved by the ethics committee of the University of Ulm (Ethikkommission Universität Ulm, A 185/09; http://www.uni-ulm.de/einrichtungen/ethikkommission-der-universitaet-ulm.html). All cells were cultured in Dulbecco’s modified eagle medium (DMEM) containing 10% fetal bovine serum (FBS), 1% l-glutamine and antibiotics in an incubator at 37°C and 9% CO_2_. Under our conditions, 9% CO_2_ were needed to ensure a pH value of 7.4 in the culture media. For experiments, 3×10^5^ cells/cm^2^ were seeded.

### Protein Transduction

The tested reagents differ in the mechanisms of protein transduction, as described by the manufacturers. CPP based transduction: Proteoducin (Jena Bioscience, Jena, Germany), Chariot (Active Motif, Carlsbad, Ca); lipid based transduction: Pro-Ject (Pierce, Bonn, Germany), PROTEOfectene (Biontex, Martinsried/Planegg, Germany), Pro-DeliverIN (OZ Bioscience, Marseille, France), Lipodin-Pro (Abbiotec, San Diego, CA); non lipid polymer based: TransPassP (New England Biolabs, Ipswich, MA), TurboFect (Fermentas St. Leon-Rot, Germany); endosomolytic: Endo-Porter (Gene Tools, Philomath, OR). The transduction of Atto488-BSA (Jena Bioscience, Jena, Germany) was performed as described in the manufacturers’ handbooks for starting point or standard transductions. The protocols were matched in amounts of transduced protein (∼0.5 µg per 3×10^5^ cells) and transduction times (20 h) to get comparable results.

### Measurement of the Internalised Protein

The quantification of the total fluorescence per well of the transduced cells was performed in a multiwell fluorescence reader (Fluostar Omega, BMG Labtech, Ortenberg, Germany). Twenty hours post transduction of 0.25 µg Atto488-BSA per well in a 96-well plate (Nunc Germany, Langenselbold, Germany), the cells were washed twice with serum-free medium and once with PBS. Using 100 µl RIPA lysis buffer per well the cells were incubated for 10 minutes at 20°C. The lysates were homogenised by pipetting up and down five times. The fluorescence signal was measured in the multiwell reader in 96-well plates at least in 6 independent experiments.

The measurement of the number of fluorescence positive cells, the fluorescence intensity per cell and the necrotic cells was performed using a flow cytometer (Cyflow Space, Partec, Münster, Germany). The wavelength filter set for FITC/GFP detection (excitation 488 nm, BP 536/40) fits to Atto488 as well. Cells were seeded in 12 well plates (Nunc Germany, Langenselbold, Germany) and 2 µg Atto488-BSA was transduced as described. The cells were washed twice with PBS, trypsinized, centrifuged for 10 min at 120×g, washed twice again to be finally resuspended in PBS without Mg^2+^ and Ca^2+^ containing 2 µg/ml propidiumiodide to detect necrotic cells. Cells were kept on ice until the flow cytometer measurements were performed. The counting was set to 20,000 gated events. To determine the reliability of the flow cytometer measurements, untreated cells (controls) and cells transduced with Atto488-BSA using Proteoducin or Endo-Porter were tested in 4 independently performed experiments. The percentages of fluorescence positive and necrotic cells revealed small standard deviations (Figure S1).

### Photochemical Internalization

To achieve endosomal release of the internalised and entrapped fluorescing cargo, photochemical internalisation was performed posttransductionally. Twenty-four hours after seeding of 3×10^5^ cells per cm^2^ in (i) 8-well chamber slides or (ii)12-well plates the cells were incubated for 18–20 h in DMEM containing 1 µg/ml TPPS_4_ (5,10,15,20-Tetrakis-(4-sulfonato-phenyl)-21,23H-porphyrin, TriPorTech GmbH, Lübeck, Germany) [12]. Then the cells were washed twice with PBS and transduced with 0.5 µg (i) or 2 µg (ii) Atto488-BSA according to the respective manufacturers’ handbook as described above. After the transduction, the cells were washed twice with serum free DMEM and once with PBS. In the chamber slides (i), 500 µl PBS per well was added. The required light exposure for ROS-production and endosome disruption was performed with a blue light lamp containing 4 neon tubes (Osram L 18W/67, Osram, München, Germany) with 8.26 mW/cm^2^ for 200 s. Subsequently, the cells were checked for fluorescence signals either fixed for 20 minutes with 4% paraformaldehyde or stored in PBS containing 2% FBS for analysing living cells.

In the experiments using 12 well plates (ii) 2 ml DMEM per well was added. The light exposure was performed directly for about 150 s with a fluorescence microscope (Zeiss IM 35, Carl Zeiss, Jena, Germany) using a FITC/GFP filter set (BP 436/FT 460/LP 470).

### Fluorescence Microscopy

For the localisation of the fluorescence signals the sarcoma cells were examined either fixed with 4% paraformaldehyde or alive in PBS +2% FBS using fluorescence microscopy. The fluorescence filter set for FITC/GFP was used. For live imaging of the PCI treatment, the light exposure of TPPS_4_ treated and transduced sarcoma cells was performed by the invert fluorescence microscopes’ halogen lamp using a FITC/GFP filter set (150 s). Pictures were taken every 2–3 seconds. The single images were loaded to the ImageJ software (http://rsbweb.nih.gov/ij/) to create movie files. To confirm the intracellular location of the fluorescent signals a laser scanning microscope controlled by the LSM 510 Meta software (Carl Zeiss, Jena, Germany) was used to take 0.9 µm thick layer z-stacks of transduced sarcoma cells. Image analysis was performed using the LSM Image Browser (Carl Zeiss, Jena, Germany).

### Analysis of the Fluorescence Signal Distribution Before and After PCI

Endosomal escape results in an altered intracellular fluorescence signal distribution. To measure the differences in the signal distribution, 8-bit converted black and white pictures (Qicam, QImaging, Surrey, BC, Canada) of transduced sarcoma cells (n = 10) before and after PCI were compared. Single cells were marked and the distribution of the grey values of all pixels of the cell was calculated by the ImageJ software. The grey values corresponding to the fluorescence signal strength range from 0 (true black, no signal) to 255 (true white, strong signal). The median of the grey values of the single cells’ pixels was determined before and after PCI treatment as demonstrated in a scheme (Figure S2). Cells with predominantly punctual signals before PCI treatment show a vast majority of pixels with low gray values resulting in a low median of the gray values of all pixels. An overcoming of endosomal entrapment results in a spreading of signals throughout the cytosol. The number of low gray values is reduced, the number of high values is increased and the median of the values as a measure of the degree of distribution is raised. Higher medians represent a higher background contribution and therefore higher cytosolic signals. For the measurement of distribution changes in consequence of a PCI treatment, exposure times were optimized to allow a clear detection of a signal spreading. Therefore, no quantitative statements about amouts of uptaken protein can be made out of these microscope experiments.

### Measurement of Cytotoxicity and Protein Activity After Transduction and PCI Treatment

The cytotoxicity of transduction experiments (measured by detection of cell death) was determined by flow cytometry using propidiumiodide (2 µg/ml) treatment as described above. The cytotoxicity of the PCI treatment was measured by trypanblue treatment. The transduced and PCI treated sarcoma cells were trypsinized and taken up in 1 ml DMEM 3 h, 24 h and 48 h after PCI treatment. Trypanblue 0.5% (250 µl) was added, incubated at 20°C for 3 minutes and the cells were screened for blue (necrotic) cells using a Neubauer-chamber. Each experiment was performed in 3 independent approaches (n = 300−900 counted cells).

To test the functionality of a transduced protein after a PCI treatment, 1 µg of a functional subunit (119 kDa) of β-galactosidase was transduced into sarcoma cells using the Chariot transduction reagent in 12-well plates. Following PCI treatment, the cells were checked for β-galactosidase activity using an X-Gal staining kit (Genlantis, San Diego, CA) according to the manufacturers handbook.

### Statistics

For statistical analysis, student’s t-tests were performed. Error bars represent either SDs or 95% confidence intervals as indicated in the figure legends. A p-value ≤0.01 was termed as significant (p≤0.01 = *, p≤0.001 = **, p≤0.0001 = ***).

## Results

### Transduction Protocols Result in Varying Amounts of Transduced Atto488-BSA and Cytotoxicity

**Figure 1:**
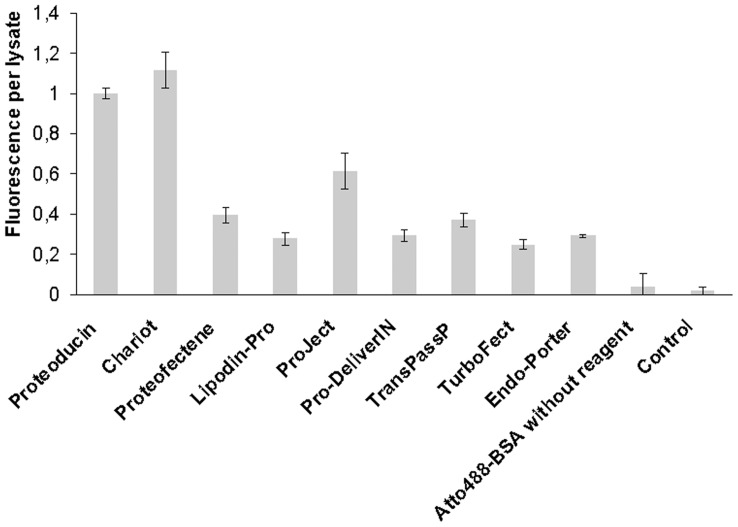
Fluorescence signal per lysate after transduction. The fluorescence intensity of the lysed cells per well was measured in a multiwell fluorescence reader. The intensities were normalized to the mean intensity produced by the transfection using Proteoducin as transduction reagent. The sarcoma cells were transduced with Atto488-BSA using different transduction reagents. Untreated sarcoma cells were measured as controls. As a control for standard endosomal uptake, sarcoma cells were treated with DMEM containing 0.5 µg Atto488-BSA without a transduction reagent (termed as Atto488-BSA). Error bars represent the 95% confidence intervals of the means. Each transduction reagent was tested in at least 6 independent experiments.

**Figure 2:**
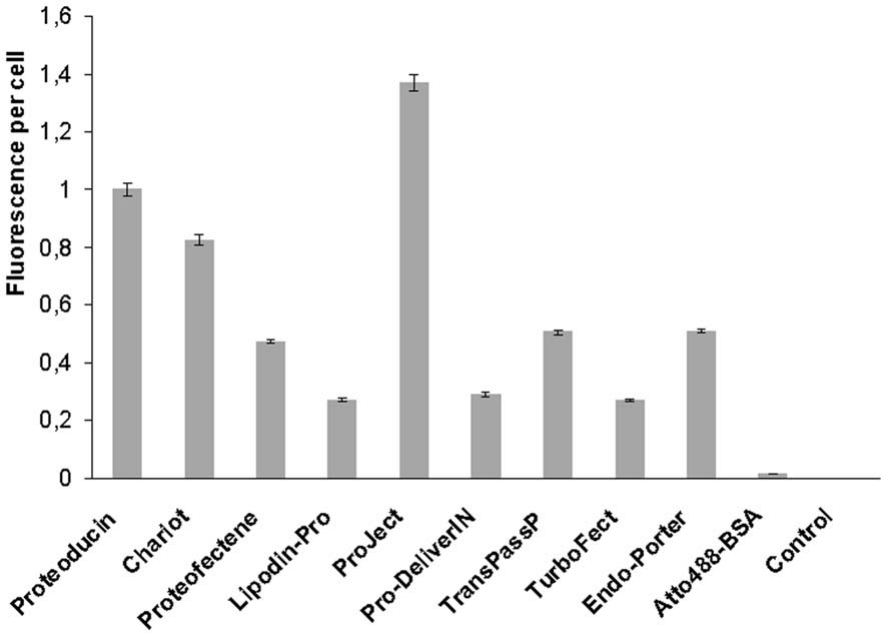
Fluorescence signal per cell after transduction. Sarcoma cells were transduced with Atto488-BSA using different transduction reagents. The fluorescence was measured in a flow cytometer, untreated cells were used as control and standard endocytosis was measured by treating the cells with 2 µg Atto488-BSA containing DMEM. Error bars represent the 95% confidence intervals of the means depicted. Measurements were set to 20000 gated events.

The transduction efficiency is often measured as the total fluorescence intensity of cell lysates after transduction of a labelled protein measured by a fluorescence reader. In our experiments, these fluorescence intensities varied in the sarcoma cell lysates of Atto488-BSA treated cells depending on the used transduction reagents (Figure 1). The highest intensities were found in cells transduced with Proteoducin, ProJect or Chariot. These differences are not due to the percentage of fluorescence positive cells detected in flow cytometry (Figure S3 A, C). Comparing the different transduction reagents, the percentage of fluorescence positive cells varied between 89.2 (TurboFect) and 96.8% (Endo-Porter). The differences in total fluorescence intensity between the transduction protocols are related to differences in the fluorescence intensity per transduced cell (Figure 2). Transduction with Proteoducin, ProJect and Chariot resulted in the highest fluorescence signals per cell. The intercellular variation in intensities was found to be very low with all protocols. The uptake rate by standard cellular endocytosis was tested by adding Atto488-BSA to the cell culture medium without any transduction reagent. Very low cellular signals were detected.

The cytotoxicity of the transduction protocols differed as shown in flow cytometer measurements in cells stained with cell death detecting propidiumiodide (PI) (Figure S3 B). The relative number of necrotic cells increased in the cells treated with TransPassP, ProJect or TurboFect. Cells treated according to the other transduction protocols showed no increase of necrotic cells. A correlation of the cytotoxicity with the amount of transduced protein measured above could not be found. The transduction efficiency tests show striking differences in the amount of transduced Atto488-BSA into the cells and cytotoxicity although the percentage of transduced cells is comparable in the experiments using different transduction reagents.

### Transductions Result in Predominantly Punctual Localisation of the Transduced Fluorescent Proteins

The localisations of the Atto488-BSA signals were checked by confocal fluorescence microscopy directly after protein transduction into the sarcoma cells. All tested transduction protocols resulted in a predominantly punctual signal distribution, often with a perinuclear concentration (Figure S4). Z stacks and a laser scanning recording a layer thickness of 0.9 µm confirmed that the signals were derived from intracellular locations (Figure S5). This suggests that the fluorescent proteins were taken up by endocytosis and remain trapped to a large extent inside the endo-lysosomal pathway. Only the Endo-Porter reagent produced a detectable homogeneous fluorescence throughout the cell (suggesting cytosolic localisation), which, however, was rather low when compared to the punctate signals (Figure S4).

To test whether the endosomal uptake was an effect of the used protein or cell line, we transduced primary human fibroblasts (FP1) with the Atto488-BSA and the sarcoma cells with an FITC-labelled anti-actin antibody. Both experiments lead to comparable punctate fluorescent signals in the respective cells (Figure S6). In summary, in all tested protocols the vast majority of transduced proteins remained trapped in intracellular punctual structures.

### Escape of Entrapped Proteins during PCI Treatment

**Figure 3:**
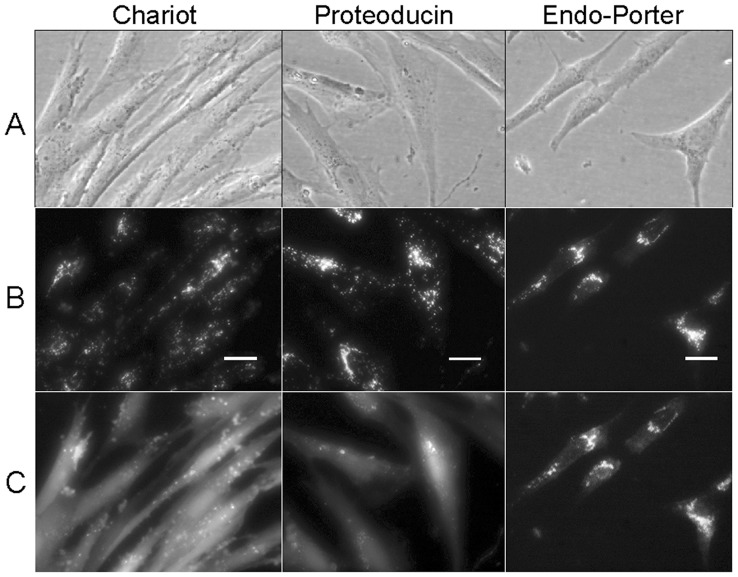
Effects of PCI of transduced Atto488-BSA in sarcoma cells. (A) Phase contrast picture of living sarcoma cells after protein transduction using the transduction reagents Chariot, Proteoducin or Endo-Porter. (B) Fluorescence signals inside of the same sarcoma cells. The signals are predominantly punctate with a perinuclear localisation. Only in Endo-Porter transduced cells a cytosolic signal is visible. (C) Fluorescence signals of the same cells after 200 seconds blue light exposure. The Atto488 signal has spread throughout the cytosol. In cells transduced via Chariot and Proteoducin, a clear fluorescence distribution change is visible. The existing cytosolic signal in Endo-Porter transduced cells is increased although the effect is weaker compared to the other reagents.

As a pilot test to deliver previously transduced and endo-lysosomal entrapped Atto488-BSA to the cytosol, the protein transduction approaches utilizing Chariot, Proteoducin or Endo-Porter were combined with a PCI treatment using the photosensitizer TPPS_4._ The cells were investigated by fluorescence microscopy. During the illumination with blue light for 180 seconds corresponding to the excitation maximum of the photosensitizer, the Atto488 signals spread throughout the cytosol, especially in cells transduced via Chariot and Proteoducin (Figure 3). Movie S1 shows the PCI-mediated overcoming of endosomal entrapment in Atto488-BSA transduced (via Proteoducin) sarcoma cells in fast motion.

### Comparison of the PCI Induced Release of the Entrapped Protein Using Different Transduction Reagents

**Figure 4:**
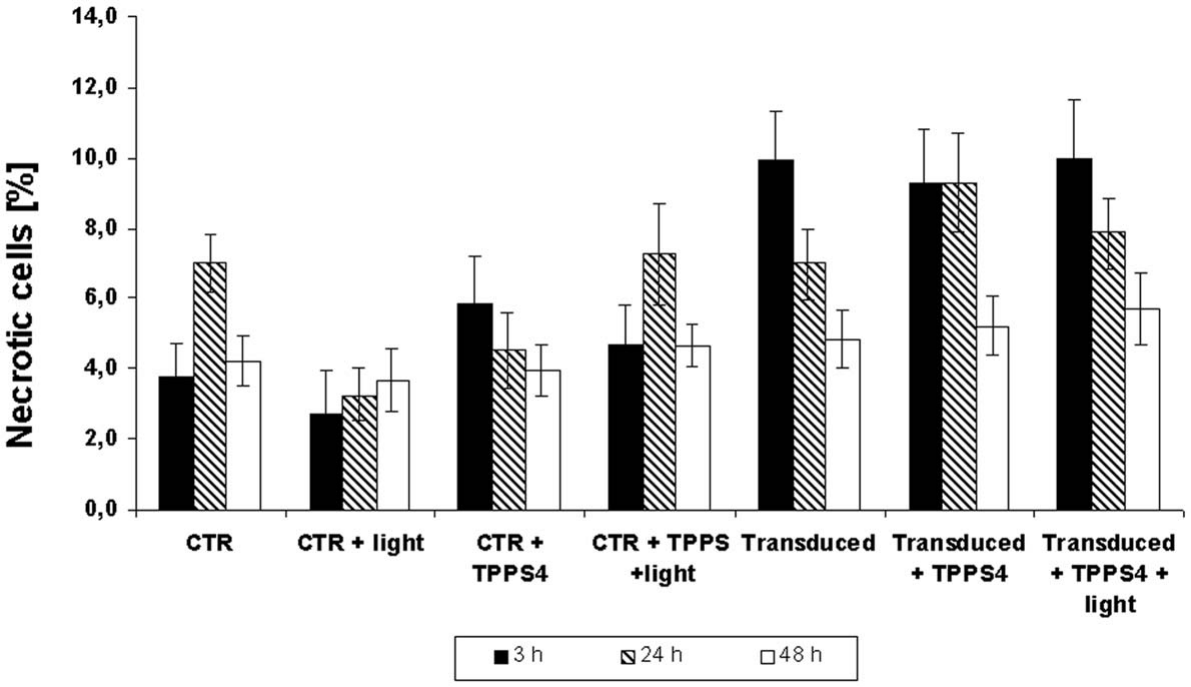
Cytotoxicity of the PCI treatment. To measure cytotoxicity of the PCI application sarcoma cells were stained with trypan-blue 3 h, 24 h and 48 h after various treatments. Cells exposed to light (L), TPPS_4_ (PS) or with both show no striking changes in the percentage of trypanblue positive cells compared to untreated controls. Cells transduced with Proteoducin (T) show an initial increase of dead cells. Additional treatments with the TPPS_4_ photosensitizer or complete PCI did not change the percentage of trypanblue positive cells. Each experiment was performed 3 times (n = 300−900). The error bars represent the 95% confidence intervals of the means.

We tested whether the effects of PCI treatments were similar in the different transduction experiments. Because PCI treatments may harm cells by damaging the cellular membrane, the cytotoxicity was tested using a trypanblue assay. The experiments revealed that PCI treatment did not increase the levels of necrosis in the sarcoma cells in the tested time frame (Figure 4). Neither the photosensitizer nor the blue light illumination nor the combination of both (complete PCI treatment) increased the cytotoxicity significantly compared to non-transduced controls or in Atto488-BSA transduced cells. An initial cytotoxic effect from about 4% to 10% of the cells was due to the transduction itself and was diminished to cell death rates similar to those of the controls within 2 days. This points to a small toxic effect of the transduction itself without long term toxicity because initial increased cell death rates decrease with population doublings whereas long term toxic effects result in consistent or increased cell death rates.

**Figure 5:**
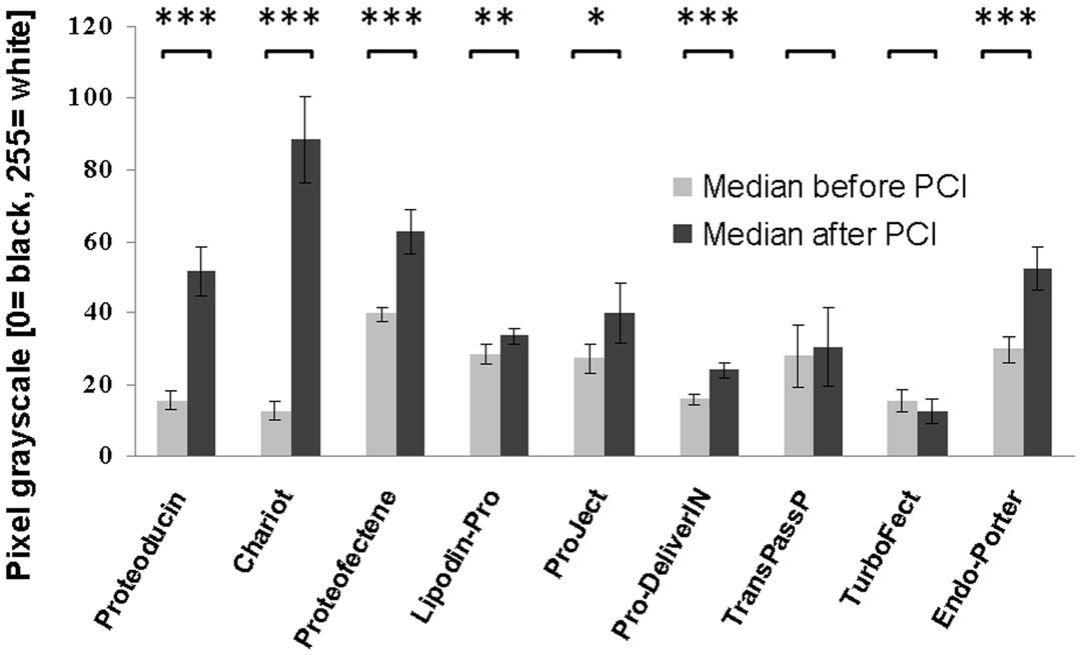
Change of signal distribution after PCI treatment. The median of the pixel gray values was calculated in fluorescence images of Atto488-BSA transduced sarcoma cells using different transduction reagents. Single cells were defined as region of interest in the ImageJ software and the median of the gray values was compared between cells before and after PCI treatment as described in Methods. The means of the medians of the gray values before and after PCI treatment are given. Error bars represent the 95% confidence intervals (n = 10). CPP-based transduction reagents (Proteoducin and Chariot) show high signal distribution changes. Reagents based on lipids (Proteofectene, Lipodin-Pro, ProJect and Pro-DeliverIN) also show changes but with partially lower significance. Reagents described as non-lipid or cationic polymers (TransPassP and TurboFect) did not lead to a signal spreading as a reason of a PCI treatment. The cytosolic signals produced by transduction with the endosomolytic reagent Endo-Porter was increased significantly by further disruption of endo- and lysosomes by PCI treatment.

Experiments using a combination of PCI treatments with 9 different transduction protocols were performed. The intracellular fluorescence distribution changes were measured as change in the medians of the distribution of the cellular pixel greyscales as described in the methods section. In 7 out of 9 transduction protocols, PCI treatment resulted in a significant change of the distribution of the Atto488-BSA signals (Figure 5). Reagents based on CPPs (Proteoducin, Chariot) showed a particularly strong and highly significant (p = 2.03*10^−6^ and p = 5.20*10^−7^) cytosolic distribution after PCI treatment. Lipid based transduction reagents (Proteofectene, Lipodin-Pro, ProJect, Pro-DeliverIN) showed less prominent effects. Reagents described as non-lipid or cationic polymers (TransPassP, TurboFect) showed no or only slight changes of the signal distribution after PCI treatment. Transduction via the endosomolytic reagent Endo-Porter produced *per se* some cytosolic signals which increased following additional PCI treatment. Thus, the capability of overcoming endosomal entrapment appears to be influenced by the mechanisms employed to induce the protein uptake.

To test whether the internalised proteins are still active after transduction and PCI treatment the 119 kDa subunit of β-galactosidase was transduced into sarcoma cells using the Chariot transduction reagent followed by PCI treatment. The treated cells showed β-galactosidase activity indicating that the combination of transduction and PCI does not inactivate the internalised proteins. β-galactosidase activity was clearly detectable in transduced and PCI treated cell whereas not transduced, PCI treated cells revealed undetectable β-galactosidase activity (Figure S7).

## Discussion

The major limitation of several protein transduction protocols is the endosomal entrapment of the transduced proteins. In this study we tested a novel approach to overcome this problem: the combination of transduction protocols and PCI in cultured cells. We found that transduction protocols differ in the mechanisms of transduction and influence the results of PCI. We tested the approach using 9 commonly used and commercially available non-covalently binding protein transduction systems. A nontoxic fluorescently labelled protein of medium size (Atto488-BSA) was selected as cargo. Without PCI, as expected all reagents induced protein uptake in a high percentage of cells, but predominantly endosomal signals were found in 8 out of 9 protocols. All transduction protocols revealed low intercellular variations with respect to the transduced protein. Therefore, it can be assumed that comparable amounts of protein reached the individual cells in the different transduction experiments. This uniformity of transduction is important in terms of applications *in vivo*. Differences between the transduction reagents were found in the intensities of cellular fluorescence per cell correlating with the amount of transduced protein; as well as in the degrees of toxicity, independent of the amounts of transduced protein. These findings can be important in experiments when only small amounts of a protein is available e.g. due to limitations in the production or purification of the protein. The unexpected differences in cytotoxicity measured in sarcoma cells may be cell type specific.

All transduction protocols preferentially revealed intracellular punctate fluorescence signals. A primarily endosomal uptake of proteins is consistent with the observation that cytoplasmic signals could only be observed in Endo-Porter treated cells. The Endo-Porter reagent is a specialized reagent to disrupt endosomal membranes as a consequence of endosomal acidification and it is the only one of the reagents tested which has exclusively been invented to overcome endosomal entrapment.

The combinations of the 9 transduction protocols and PCI were tested for a homogeneously distributed localization of a transduced marker protein using a newly developed method of cell image analysis, which is able to spatially allocate signal intensities. The used PCI is a fast and reliable technique using a nontoxic photosensitizer and common fluorescence microscopy, making this technique usable with easily accessible equipment and reagents.

PCI is based on the production of reactive oxygen species by a photosensitizer, in our experiments TPPS_4._ We chose this photosensitizer because of its documented functionality, its excitation maximum fitting to the FITC/GFP filters [13] and its convenient accessibility. After light induced disruption of the endosomal membranes [12] the entrapped proteins are released to the cytosol. Therefore, PCI *per se* can have toxic effects on cells. Along this line it is interesting to note that PCI has originally been developed from the photodynamic therapy (PDT) that is often used in anti-cancer treatments. Here, cells are incubated with photosensitizers and higher light dosages are used [18]. The acute production of reactive oxygen species leads to cell death as a result of a destabilization of large portions of the cellular membrane. Hence, for successful PCI without inducing cell death, the phototoxicity has to be assessed precisely for the cell type under investigation. Our PCI protocol is nontoxic to the sarcoma cells utilized in this study. Our combination experiments show that the disruption of endosomes and lysosomes by PCI does not evenly affect signal distribution in all transduction protocols tested. This points to different ways of induced uptake regarding the differing transduction reagents. Best results were obtained using two CPP related transduction protocols. The protein uptake directed by CPPs seems to be preferentially endosomal. Subsequent to PCI, a disruption of endosomes and lysosomes induces a clear signal distribution change from punctual to uniform background or cytosolic signals. Remaining punctate signals in experiments using lipid shuttle systems can be possibly attributed to a failure of a fusion of the shuttle reagents with membranes. Endosomally trapped lipid shuttles would create punctate signals. Disruption of endosomal membranes would free the shuttles but the proteins would stay enriched and trapped inside of the lipid shuttles that float through the cytosol and the signals would remain punctual. This problem could possibly be solved by addressing the lipid shuttles with photosensitizers, too. Using this approach the illumination of the sensitizers could disrupt both the endolysosomal vesicles and the lipid shuttles. The punctual signals produced by the tested transduction reagents classified as non-lipid polymers do not change significantly after a PCI treatment. This may be the result of a strong binding of those reagents both to the protein cargo and to the cellular membrane. Therefore an endo-lysosomal disruption would not lead to a spatial signal distribution because the proteins would remain attached to endo-lysosomal membranes independent of the integrity of the organelles.

Huge amounts of internalized cargo pass transduced cells through the endo-lysosomal pathway without reaching the functional destination location if no endosomolytic treatment is used. Keeping this in mind, endosomolytic treatments like PCI bear interesting possibilities in *in vitro* and *in vivo* experiments. Recently, experiments aiming at the generation of induced pluripotent stem cells (IPSCs) using CPP-mediated protein transductions were performed [19]. Although a reprogramming of fibroblasts was successful after adding the reprogramming enhancement reagent valproic acid, low transduction efficiencies seemed to be a major problem [20]. Combining the transduction of CPP-fusion proteins and PCI may enhance the IPSC colony yield several fold.

Diseases that are caused by genetic defects of a single gene and therefore by a reduction of a functional protein could be treated by low dose uptake of functional protein in all body cells. For such an approach it is very important to keep the needed amount of protein reaching the cells minimal for that medication shuttles are limited in size. In addition, an overdose of protein circulation in a body may have toxic effects in a patient. Reminding this, the optimization of proteins reaching the cytosol might be the crucial factor in protein transduction treatments.

### Concluding Remarks

In protein transduction experiments, one of the most important factors is to translocate the protein of choice to its destination location, frequently the cytoplasm. The entrapment of transduced proteins within the endo-lysosomal pathway is the critical step after the induced uptake into the cells. Our study shows that a combination of cell penetrating peptide mediated protein uptake and PCI should be the first consideration in these experiments because it provides both a high uptake rate into cells and a high translocation capability from vesicles to the cytosol.

## Supporting Information

Figure S1 **Reliability of the Flow cytometer measurements.** Atto488-BSA transductions were performed with 2 randomly chosen transduction reagents (Proteoducin and Endo-Porter) in 4 independent experiments. Additionally transductions with a third reagent (TurboFect) were performed in 2 independent experiments. As control, 4 separately cultured untreated sarcoma cells were used. (A) Measurement of the necrosis and late apoptosis by propidiumiodide treatment. The variations within the multiple tests were small. Error bars represent +/− SD. Proteoducin and Endo-Porter transduced cells show no increase of necrotic or late apoptotic cells whereas TurboFect transduced cells show a clear increase of propidiumiodide positive cells. (B) Percentage of the transduced cells. Multiple tests of the same transduction approaches revealed extremely small variations (Error bars = +/−SD). Therefore all transduction reagents were very stable in the transduction efficiency.
(TIF)

Figure S2 **Scheme of the procedure to determine the signal distribution.** (A) Phase contrast of Atto488-BSA transduced (via Proteoducin) sarcoma cells (1). The yellow line defines a cell as region of interest (ROI). Overlay of the ROI in fluorescence pictures of the same Atto488-BSA transduced cells either (2) before or (3) after PCI treatment. (B) Intensity count histograms of the pixels inside the ROI. The peaks represent the cytosolic fraction for that the puncate endosomal signals are numerically few compared to the dark cytosolic pixels. The median pixel intensity is given for both intensity counts. In transduced but not PCI treated cells (blue line), most pixels have low signal intensities resulting in a median signal intensity (measured as grey scale value) of 12. After PCI treatment of the same cells (red line) the pixels with higher signal intensities are increased leading to an increased median signal intensity of 56. Magnification: The pixel count of high signal intensities representing strong endosomal signals are shown before and after PCI treatment. The disappearance of most of the high intensity pixels after the PCI treatment points to a disruption of the endosomal vesicles.
(TIF)

Figure S3 **Comparison of transduction rates, cytotoxicity and measured levels of transduced proteins using nine different transduction reagents.** Whereas the percentage of transduced cells is quite homogeneous in all transduction protocols (A), the amount of transduced protein (measured by fluorescence intensity) shows striking differences (C) which can not be attributed to the amount of necrotic cells (B) as well. (A) The transduction rates are high in all used transduction protocols. The percentages of transduced cells differ slightly ranging from 89.2% (TurboFect) to 96.8% (Endo-Porter) (n = 20000). (B) Percentage of necrotic cells after transduction measured by propidiumiodide staining. Only 3 transduction reagents (ProJect, TransPassP and TurboFect) induced considerable increases in cell death rates compared to untransduced controls. The relatively high cell death rates of untransduced controls are due to the repeated washing steps previous to the flow cytometer measurements. (C) Fluorescence intensity of lysed cells per well measured in a multiwell fluorescence reader. The intensities were normalized to the mean intensity produced by the transduction using Proteoducin as transduction reagent. Error bars represent the 95% confidence interval of the means. Each transduction reagent was tested in at least 6 independent experiments.
(TIF)

Figure S4 **Transduction of Atto488-BSA using different transduction reagents.** All reagents produced punctual Atto488 signals within the sarcoma cells which were often located close to the nucleus. Only in cells transduced by Endo-Porter, a low level homogeneous fluorescence signal throughout the cell was detectable, suggesting cytosolic localisation of Atto488-BSA. Scale bars represent 10 µm.
(TIF)

Figure S5 **Intracellular localisation of the internalized Atto488-BSA.** Proteoducin Atto488-BSA transduced sarcoma cells were recorded by confocal laserscanning microscopy (0.9 µm layer) confirming that the signals were inside the cells. Shown is the phase contrast (PH), the fluorescence signals (FL) and the merged pictures (Merge).
(TIF)

Figure S6 **Punctuated protein localization in fibroblasts and neurofibrosarcoma cells detected by fluorescence microscopy.** Phase contrast is shown in blue color, the Atto488−/FITC- signal in green color. (A) Primary fibroblasts (FP1) were Atto488-BSA transduced using the Proteoducin reagent. The signals are punctate with a predominated perinuclear location. (B) Transduction of a FITC-labeled anti-actin antibody into neurofibrosacoma cells produced comparable signals.
(TIF)

Figure S7 **β-galactosidase transduced cells show β-gal activity after PCI treatment.** β-gal transduced, PCI treated neurofibrosarcoma cells are shown (A) in phase contrast and (B) as a bright field picture. Untransduced, PCI treated neurofibrosarcoma cells are given (C) in phase contrast and (D) as a bright field picture. All cells were stained for β-gal activity. The transduction was performed using the Chariot transduction reagent. The β-gal transduced and PCI treated cells show a clear β-gal activity (A+B) whereas no β-gal activity could be detected in the untransduced and PCI treated cells (C+D).
(TIF)

Movie S1 **Live movie of the escape of the Atto488 signals in living cells.** The movie shows the overcoming of endosomal entrapment during PCI treatment. Transduced and TPPS_4_ treated sarcoma cells were illuminated using a FITC/GFP filter set. The light reaching the cells excites both the photosensitizer TPPS_4_ and the Atto488. Therefore pictures could be taken throughout the whole PCI treatment. Single images were put together as an image sequence and converted into avi format. 1 second of the movie represents 6 seconds of the treatment.
(AVI)
