# Supplementary figures and images for: Enhancing Endosomal Escape of Transduced Proteins by Photochemical Internalisation
Source: PLoS One. 2012 Dec 21;7(12):e52473. doi: 10.1371/journal.pone.0052473 (PMC3528648; doi:10.1371/journal.pone.0052473)

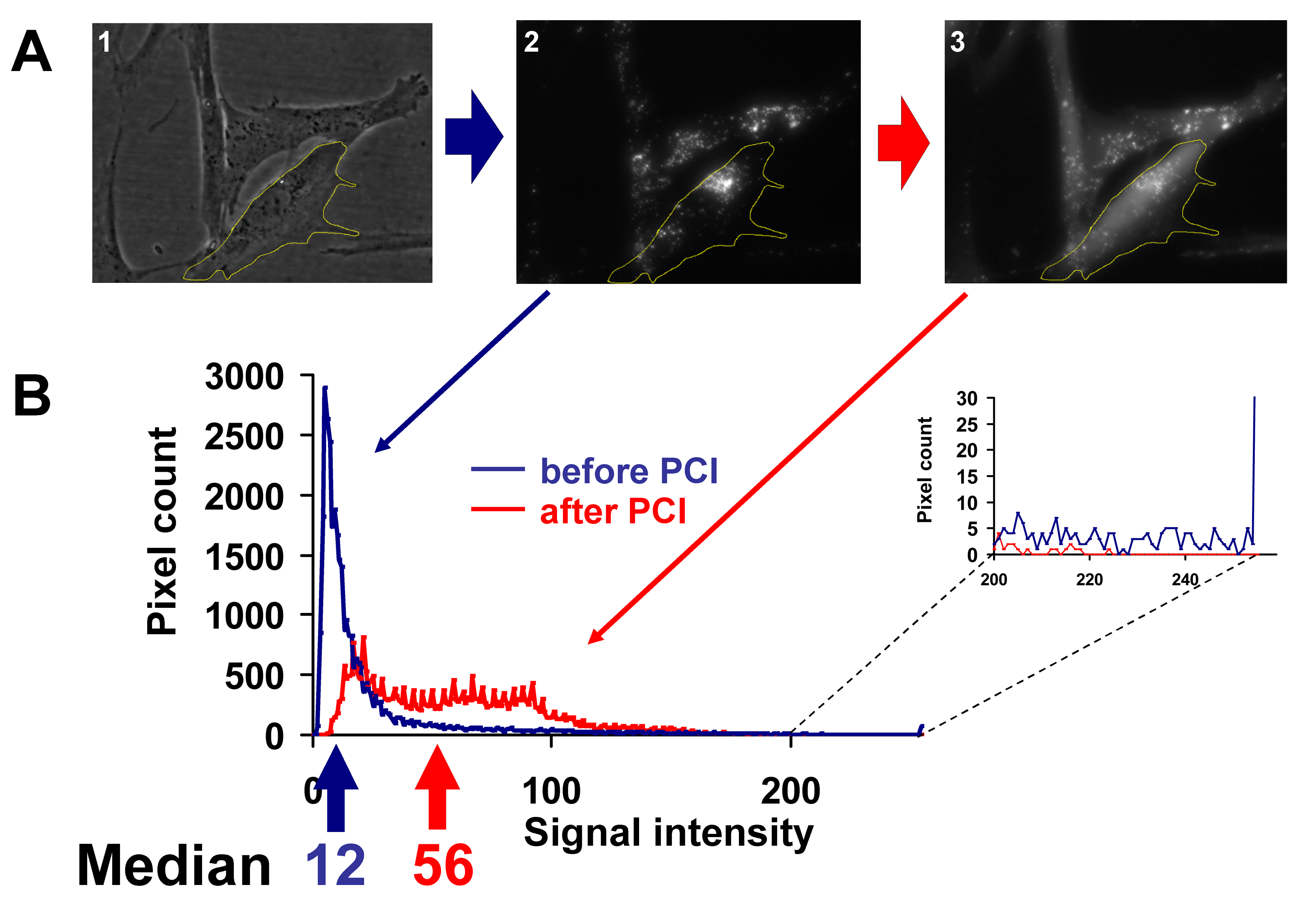

Supplement: Figure S2 — Scheme of the procedure to determine the signal distribution. (A) Phase contrast of Atto488-BSA transduced (via Proteoducin) sarcoma cells (1). The yellow line defines a cell as region of interest (ROI). Overlay of the ROI in fluorescence pictures of the same Atto488-BSA transduced cells either (2) before or (3) after PCI treatment. (B) Intensity count histograms of the pixels inside the ROI. The peaks represent the cytosolic fraction for that the puncate endosomal signals are numerically few compared to the dark cytosolic pixels. The median pixel intensity is given for both intensity counts. In transduced but not PCI treated cells (blue line), most pixels have low signal intensities resulting in a median signal intensity (measured as grey scale value) of 12. After PCI treatment of the same cells (red line) the pixels with higher signal intensities are increased leading to an increased median signal intensity of 56. Magnification: The pixel count of high signal intensities representing strong endosomal signals are shown before and after PCI treatment. The disappearance of most of the high intensity pixels after the PCI treatment points to a disruption of the endosomal vesicles. (TIF) [file pone.0052473.s002.tif]

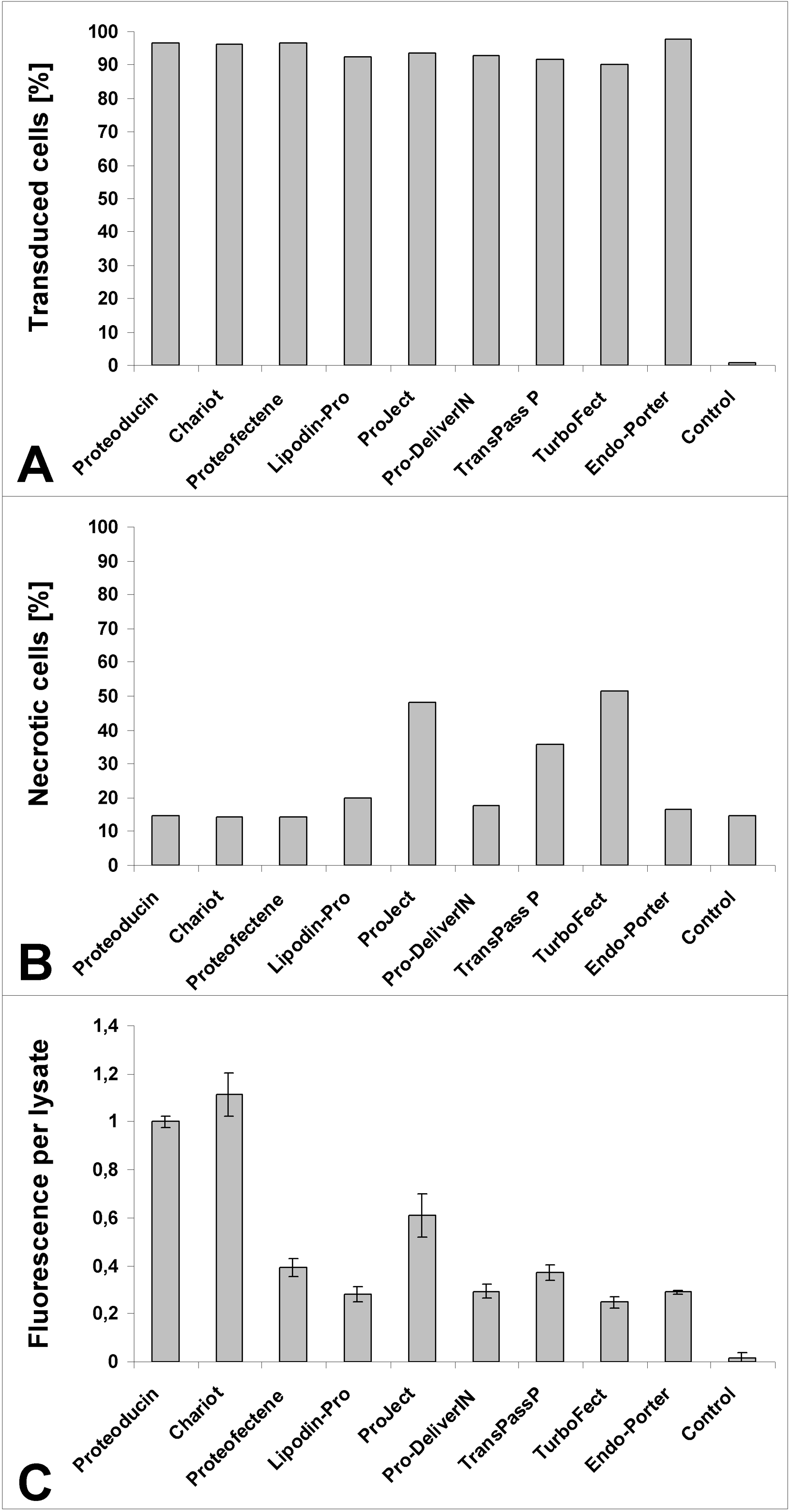

Supplement: Figure S3 — Comparison of transduction rates, cytotoxicity and measured levels of transduced proteins using nine different transduction reagents. Whereas the percentage of transduced cells is quite homogeneous in all transduction protocols (A), the amount of transduced protein (measured by fluorescence intensity) shows striking differences (C) which can not be attributed to the amount of necrotic cells (B) as well. (A) The transduction rates are high in all used transduction protocols. The percentages of transduced cells differ slightly ranging from 89.2% (TurboFect) to 96.8% (Endo-Porter) (n = 20000). (B) Percentage of necrotic cells after transduction measured by propidiumiodide staining. Only 3 transduction reagents (ProJect, TransPassP and TurboFect) induced considerable increases in cell death rates compared to untransduced controls. The relatively high cell death rates of untransduced controls are due to the repeated washing steps previous to the flow cytometer measurements. (C) Fluorescence intensity of lysed cells per well measured in a multiwell fluorescence reader. The intensities were normalized to the mean intensity produced by the transduction using Proteoducin as transduction reagent. Error bars represent the 95% confidence interval of the means. Each transduction reagent was tested in at least 6 independent experiments. (TIF) [file pone.0052473.s003.tif]

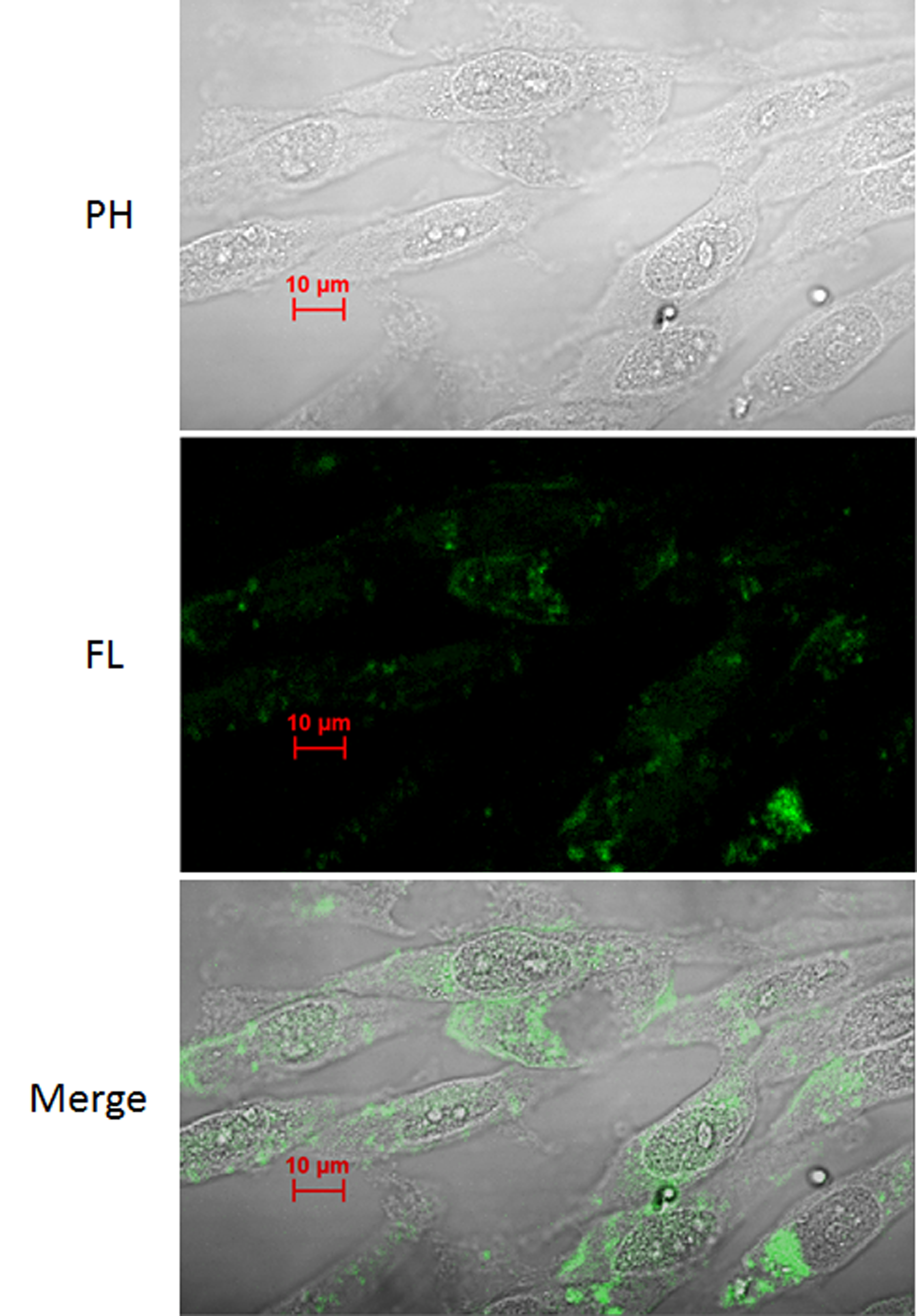

Supplement: Figure S5 — Intracellular localisation of the internalized Atto488-BSA. Proteoducin Atto488-BSA transduced sarcoma cells were recorded by confocal laserscanning microscopy (0.9 µm layer) confirming that the signals were inside the cells. Shown is the phase contrast (PH), the fluorescence signals (FL) and the merged pictures (Merge). (TIF) [file pone.0052473.s005.tif]

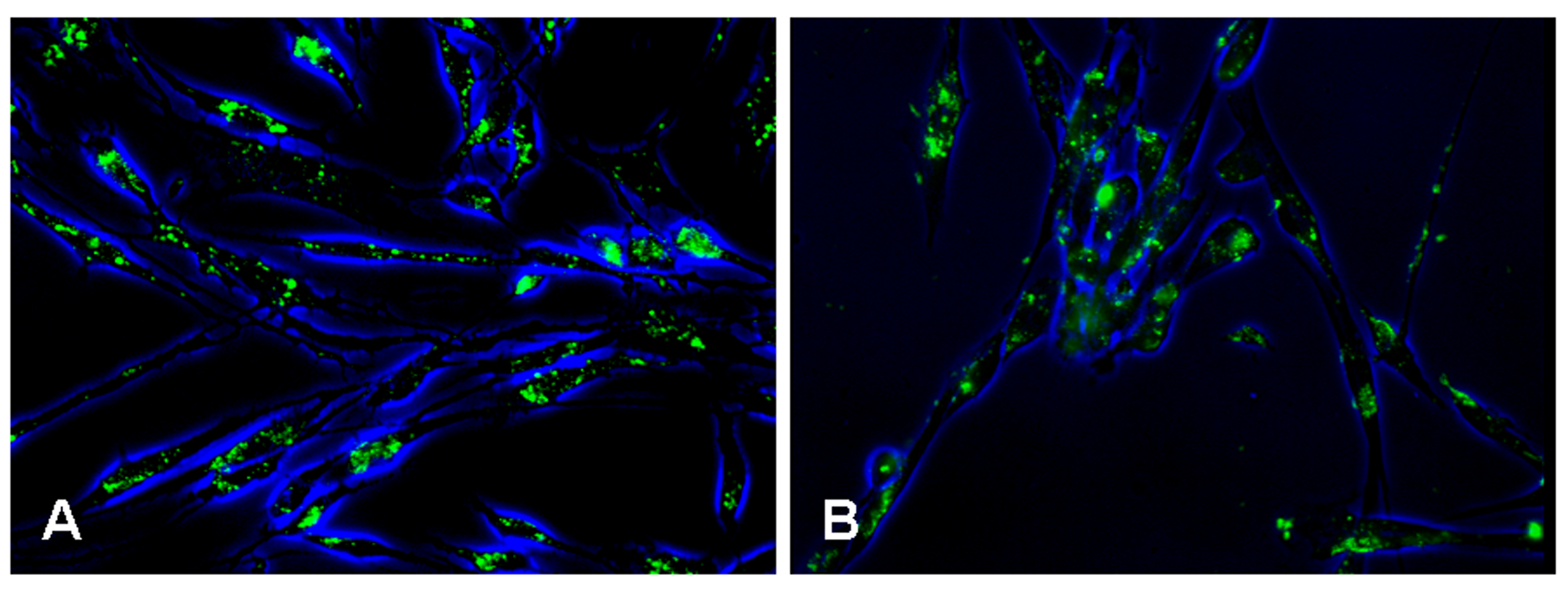

Supplement: Figure S6 — Punctuated protein localization in fibroblasts and neurofibrosarcoma cells detected by fluorescence microscopy. Phase contrast is shown in blue color, the Atto488−/FITC- signal in green color. (A) Primary fibroblasts (FP1) were Atto488-BSA transduced using the Proteoducin reagent. The signals are punctate with a predominated perinuclear location. (B) Transduction of a FITC-labeled anti-actin antibody into neurofibrosacoma cells produced comparable signals. (TIF) [file pone.0052473.s006.tif]

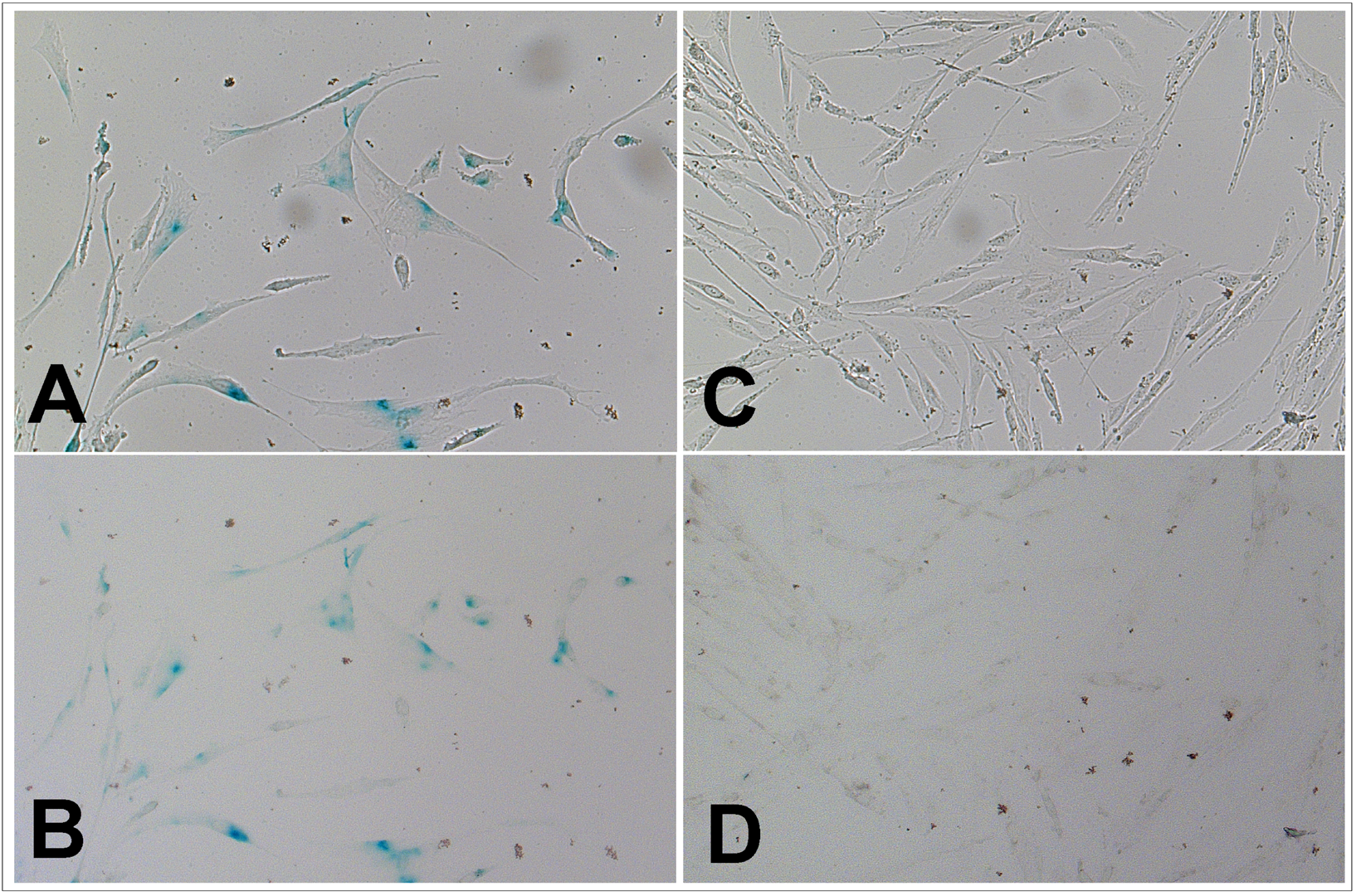

Supplement: Figure S7 — β-galactosidase transduced cells show β-gal activity after PCI treatment. β-gal transduced, PCI treated neurofibrosarcoma cells are shown (A) in phase contrast and (B) as a bright field picture. Untransduced, PCI treated neurofibrosarcoma cells are given (C) in phase contrast and (D) as a bright field picture. All cells were stained for β-gal activity. The transduction was performed using the Chariot transduction reagent. The β-gal transduced and PCI treated cells show a clear β-gal activity (A+B) whereas no β-gal activity could be detected in the untransduced and PCI treated cells (C+D). (TIF) [file pone.0052473.s007.tif]
